# Regulator Network Analysis of Rice and Maize Yield-Related Genes

**DOI:** 10.3389/fcell.2020.621464

**Published:** 2020-12-03

**Authors:** Zheng Chen, Zijie Shen, Lei Xu, Da Zhao, Quan Zou

**Affiliations:** ^1^School of Applied Chemistry and Biological Technology, Shenzhen Polytechnic, Shenzhen, China; ^2^Institute of Fundamental and Frontier Sciences, University of Electronic Science and Technology of China, Chengdu, China; ^3^School of Electronic and Communication Engineering, Shenzhen Polytechnic, Shenzhen, China

**Keywords:** crop yield, gene function, genetic network, phylogenetic, rice, maize

## Abstract

Rice and maize are the principal food crop species worldwide. The mechanism of gene regulation for the yield of rice and maize is still the research focus at present. Seed size, weight and shape are important traits of crop yield in rice and maize. Most members of three gene families, APETALA2/ethylene response factor, auxin response factors and MADS, were identified to be involved in yield traits in rice and maize. Analysis of molecular regulation mechanisms related to yield traits provides theoretical support for the improvement of crop yield. Genetic regulatory network analysis can provide new insights into gene families with the improvement of sequencing technology. Here, we analyzed the evolutionary relationships and the genetic regulatory network for the gene family members to predicted genes that may be involved in yield-related traits in rice and maize. The results may provide some theoretical and application guidelines for future investigations of molecular biology, which may be helpful for developing new rice and maize varieties with high yield traits.

## Introduction

It has been predicted that crop yields must double to meet the demands of the rising world population by 205 (Ray et al., 2013). However, it is difficult to increase the effective cultivated area, and increasing crop yield is the only way to ensure food supply. Rice and wheat are key food crops and have been the most widely consumed staple foods in most parts of the world. They are grown as annual grain and belong to the monocotyledonous grass family. At the same time, rice and maize are model crops studied in the fields of genetics and genomics of grasses. Grain yield is a complex trait multiplicatively determined by several component traits. The number of grains per panicle, panicle number per hull, and grain weight are common traits of rice and maize (Wang and Li, 2011; Bommert et al., 2013; Yang et al., 2018; Harrop et al., 2019). *ZmGS5* as the vital gene to increase the grain weight and cell number in the transgenic plants of *Arabidopsis thaliana L*., suggesting that *ZmGS5* may have a conserved function among different plant species that affects seed development (Liu et al., 2015a). It is shown that yield-related QTLs are conserved between maize and rice (Liu et al., 2017). Another yield-related QTL is kernel row number QTL, *KRN4*, which can enhance grain productivity by increasing KRN per ear (Liu et al., 2015c). The increasing of endosperm also plays a role in the crop yield, therefore, *ZmGE2* gene has effect on the maize yield, which is associated with increase in embryo to endosperm ratio in maize (Zhang et al., 2012). The analysis of key genes for crop yield traits and their genetic regulatory networks is a scientific problem that must be solved for the improvement of crop yields. APETALA2/ethylene response factor (AP2/ERF), auxin response factors (ARFs) and *MADS* genes are key factors in grain yield traits and crop domestication (Harrop et al., 2019; Li et al., 2019; Wang et al., 2019).

The AP2/ERF superfamily contains key regulators in various pathways for development and yield in plants (Riechmann and Meyerowitz, 1998). For example, *ZmRAP2.7* and *ZmEREB94*, AP2/ERF members, participated in root development and starch synthesis, respectively (Li et al., 2017, 2019). The function of *ids1/Ts6* is the regulation of spikelet pair meristem development (Wang et al., 2019). Several AP2-like genes are key factors with respect to inflorescence branching and architecture in domesticated rice (Harrop et al., 2019). *OsGL6* is involved in trichome formation in rice (Xie et al., 2020). ARFs can bind to auxin response DNA elements (AuxRE) of the genes to regulate plant development and growth (Li et al., 2016). Genes with MADS-box, a conserved sequence motif, can encode the transcription factors regulating various processes such as seed and flower development and organ differentiation in plants (Schwarz-Sommer et al., 1990; Becker and Theissen, 2003; Gramzow et al., 2010; Alvarez-Buylla et al., 2019). The *ZmMADS69* allele controls maize flowering time (Liang et al., 2019). Overexpression of *zmm28* is associated with a significant increase in grain yield in maize (Wu et al., 2019). *MADS78* and *MADS79* are key regulators in rice early seed development (Paul et al., 2020). The functions of *OsMADS57* are related to plant vegetative growth in rice (Chu et al., 2019).

Until now, related research on crop yield traits focused on single gene and their upstream and downstream regulatory pathways. However, there are rarely studies pertaining to genetic control networks of multiple genes. A genetic regulatory network (GRN) is a collection of molecular regulators and is composed of nodes and edges. The nodes, namely, regulators, can be DNA, RNA, proteins or complexes of these. The edges are the functional interaction model, called regulatory relationships, which can be positive activation and negative inhibition. Regulators and their functional interactions form the backbone of the cellular machinery. The network is a mechanism for controlling morphogenesis and individual development. The characteristics of genetic control of crop yield for rice and maize development are helpful for applying the traits in crop breeding (Yan and Tan, 2019). This can promote research on the genetic basis of the formation of major crop traits, and even the theoretical basis for the in-depth understanding of the common transformation mechanism of the genetic structure of the complex traits of grasses.

**FIGURE 1:**
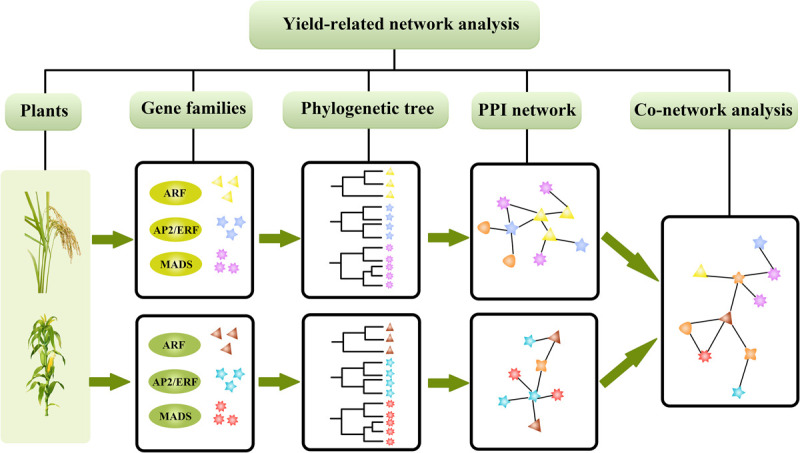
The process of analysis for genetic regulation networks in rice and maize.

As shown in Figure 1, we evaluated the evolutionary relationships of three gene families, AP2, ARF, and MADS, in rice and maize, respectively. We constructed the GRN of the gene families to predict yield-related uncharacterized genes, which can provide some theoretical guidelines for future molecular biology investigations involving high yield traits in rice and maize.

## Materials and Methods

### DATA

#### RiceData

The information of gene families related to rice is derived from RiceData^1^. This database can be used to query resources, such as excellent rice germplasms, rice mutants, molecular markers, genes, and pedigrees. Relevant literature searches were also conducted. In 2005, the Institute of Crop Sciences and Chinese Rice Research Institute of the Chinese Academy of Agricultural Sciences proposed and chaired the construction of the rice gene database. The RiceData database mainly collects genetic rice information (including QTLs), including gene names, functions, locations, and relevant references.

#### National Center for Biotechnology Information (NCBI)

National Center for Biotechnology Information was established in 1988 to build academic information systems for molecular biology (Sayers et al., 2020). The resources of NCBI comprise six categories: literature, health, genomes, genes, proteins, and chemicals (Sayers et al., 2020). In addition to biological data, NCBI also provides an assortment of analysis and visualization software (Sayers et al., 2020). We obtained sequence data for MADS and AP2 families in maize from NCBI.

### Tools

#### STRING

STRING is a web-server^2^ that is widely used to visualize data as interaction networks and to perform gene-set enrichment analysis (Szklarczyk et al., 2019). It collects and integrates known protein-protein interaction (PPI) data from all publicly available sources (Szklarczyk et al., 2019). The source databases include KEGG, Reactome, BioCyc, Gene Ontology and so on (Caspi et al., 2016; Fabregat et al., 2016; Kanehisa et al., 2017; The Gene Ontology Consortium, 2017). STRING interaction predictions are produced by computational prediction efforts, including protein co-expression systems analysis, shared genome shared signal measurement and PubMed abstracts for text mining from all databases, as well as OMIM, and so on.

#### IQ-TREE

Phylogenetic analyses have been widely used in molecular systematics. In biology, phylogenetics can be applied in the analysis of the evolutionary relationships among individuals or groups of organisms. IQ-TREE is freely available software for discovering these relationships through phylogenetic inference implementing Maximum likelihood (ML) (Nguyen et al., 2015). The substitution model was calculated with MODELFINDER (integrated in IQ-TREE; best-fit model: JTT + R5 chosen according to the Bayesian information criterion). We also constructed the phylogenetic trees for rice and maize by IQ-TREE.

#### MAFFT

Multiple sequence alignments (MSA) is widely used in the alignment of proteins and nucleotide sequences, which are assumed to be inherited from a common ancestor. Detecting co-evolution is a critical step in the prediction of protein-protein interactions (de Juan et al., 2013; Wang et al., 2017). MAFFT is MSA software that offers three alignment strategies, including the progressive method (PartTree, FFT-NS-1, and L-INS-1), iterative refinement methods (FFT-NS-i, L-INS-i, E-INS-i, and G-INS-i) and so on (Katoh and Standley, 2013; Katoh et al., 2019). We aligned protein sequences with their corresponding amino acid domains with MAFFT (Katoh and Standley, 2013; Katoh et al., 2019).

## Results

### Identification of AP2 Domain, ARF Domain and MADS Genes in Rice and Maize

The AP2 domain, ARF domain and MADS gene candidate sequences from rice and maize genomes were derived from the China Rice Data Center (see text footnote 1) and NCBI^3^. 300, 69 and 143 potential sequences were identified as AP2 domain-containing genes, ARF domain-containing genes and MADS genes, respectively. Detailed information about these genes for rice and maize are provided in Supplementary Tables 1, 2.

### Phylogenetic Analysis of AP2 Domain Proteins in Rice and Maize

**FIGURE 2:**
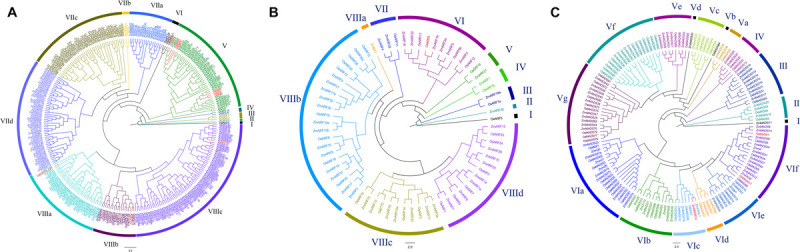
Phylogenetic trees of three gene families in rice and maize **(A)** Phylogenetic tree of the AP2 domain-containing proteins in rice and maize. Protein sequence alignment using E-INS-i algorithm. AP2 domain-containing proteins grouped into 10 subfamilies. **(B)** Phylogenetic tree of the ARF proteins in rice and maize. Protein sequence alignment using E-INS-i algorithm. Auxin response factor proteins grouped into 10 subfamilies. **(C)** Phylogenetic tree of the MADS-box proteins in rice and maize. Protein sequence alignment using E-INS-i algorithm. MADS-box proteins grouped into 17 subfamilies.

We constructed the phylogenetic tree of the AP2 protein sequences in rice and maize to illustrate the phylogenetic relationship. The phylogenetic tree for an AP2 domain-containing gene family in rice and maize revealed four major clades grouping into 10 subfamilies (Figure 2A). Among the 300 AP2 proteins, 1 belongs to group I, 1 to group II, 3 to group III, and 2 to Group IV. The large groups for AP2 members are VII and VIII. Group VII can be further clustered into four subgroups, besides three subgroups in group VIII. Group I and group II only contain one respective gene each: OsRAV2 and ZmAP2-5. Group III includes three genes of maize, and these groups are the ancient clades. From the dataset, most of the proteins containing the AP2 domain were related to crop yield (Riechmann and Meyerowitz, 1998; Harrop et al., 2019). The yield-related gene *OsEATB* is in Group VIIIb. Group V includes three close clades, with one clade containing three well-known yield-related genes, *OsRSR1*, *OsSNB*, and *OsIDS1* (Fu and Xue, 2010; Rashid et al., 2012; Lee et al., 2014; Rao et al., 2014; Ji et al., 2019). The existence of such yield-related proteins was one of the unusual features of the AP2 gene family in flowering plants, such as maize and rice. From the groups or subgroups, the crop yield-associated genes were randomly selected as representatives for further analysis. From previous research, *OsEATB* can reduce the plant height and panicle length during the maturity stage, promoting the branching potential of both tillers and spikelets (Qi et al., 2011). In rice, the absence of *BBM1*, *BBM2*, and *BBM3* would result in embryo arrest and abortion in group V (Khanday et al., 2019). Overexpression of *OsAP2-39* can cause a variety of phenotypic changes in transgenic rice, such as the reduction of plant height, tiller, leaf number and 1–2 weeks postponement for heading, ultimately resulting in a decrease in yield due to reduced biomass and grain number (Yaish et al., 2010). *OsRSR1* regulates starch synthesis in rice (Fu and Xue, 2010). Compared with the wild type, the grain size is larger and the quality and yield are higher in *rsr1* (Fu and Xue, 2010). *OsIDS1* and *OsSNB* play important roles in the establishment of inflorescence morphology and floral meristems. There is a significant decrease in branches and spikelets of the inflorescence for the double mutant *snb/osids1* plant (Lee and An, 2012). The function of the *OsSNB* gene was determined by decreased seed fall, a seed length increase of 7.0%, and a 1000-seed weight increase of 6.1% in the *ssh1* mutants (Jiang et al., 2019). The *SHAT1* gene, which encodes an AP2 transcription factor, is required for seed shattering in rice (Jiang et al., 2019).

### Phylogenetic Analysis of ARF Domain Proteins in Rice and Maize

The full-length amino acid sequences of ARF domain proteins were used for multiple sequence alignment and phylogenetic analyses, respectively. The phylogenetic tree of 69 members of ARF domain-containing genes for rice and maize was constructed (Figure 2B). These ARF domain-containing members can be distinctly divided into eight groups and 11 subfamilies. There is only one gene in groups I and II, *OsARF5* and *ZmARF20*, respectively, besides two genes confirmed in groups III, IV, and V. Group VI has 10 genes, and group VIII is the largest one of all. The gene *OsARF1* in group VIIId was indicated to be essential for growth in vegetative organs and seed development (Attia et al., 2009). Floral organ development is essential to plant yield and seed quality, so overexpression of *OsARF19/OsARF7a* resulted in high auxin content, dwarfism, shrunken grains and upregulated expression levels of *OsMADS29* and *OsMADS22*, which are two floral organ regulators (Zhang et al., 2015). OsARF2 and *OsARF4* are located in the same loci (Wang et al., 2007). During rice grain development, the interaction of *OsARF4/OsARF2* and *OsSK41* can repress the expression of some auxin responsive genes, and the grain size with respect to *osarf4/osarf2* performance is larger (Hu et al., 2018).

### Phylogenetic Analysis of MADS Proteins in Rice and Maize

To understand the evolutionary and phylogenetic relationships of MADS proteins, a phylogenetic tree using E-INS-i algorithm was constructed from rice and maize (Figure 2C). The 143 MADS protein sequences were aligned and classified into six well-supported groups and 17 subfamilies labeled with different colors. According to the phylogenetic tree, there is only one gene in group I, as well as five genes, 10 genes and four genes in groups II, III, and IV, respectively. Group V and VI are larger than others, with seven and six subfamilies, respectively. Based on previous studies, MADS protein functions are related to floral, ovule and seed organ development (Schwarz-Sommer et al., 1990; Becker and Theissen, 2003). For example, *OsMADS3*, *OsMADS13*, and *DROOPING LEAF* play various important roles in floral development (Dreni et al., 2007; Liu et al., 2011). Downregulated expression of *OsMADS7* and *OsMADS8* resulted in severe phenotype deterioration for plants, including late flowering, abnormal performance of lodicules, stamens and carpels, and a loss of floral determinacy (Cui et al., 2009). *OsMADS1* is mainly expressed in flower organs and determines the formation of the lemma and palea (Chung et al., 1994). All of the *OsMADS1* transgenic plants exhibited similar phenotypes, including dwarfism, distorted panicles, decreased numbers of branches and spikelets, and elongated sterile lemmas (Jang et al., 2017). The Gγ subunits interacting with GS3 and DEP1 can activate the expression of *OsMADS1* to regulate grain shape (Liu et al., 2018). *OsMADS17* expression is regulated by *OsMADS1* and involved in hormone signaling and floral identity (Hu et al., 2015).

### Prediction and Analysis of Genetic Network for Rice Yield-Related Genes

**FIGURE 3:**
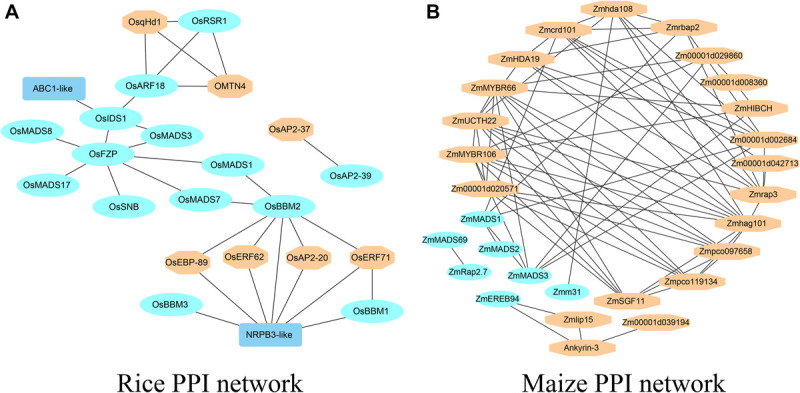
The PPI networks of yield-related genes **(A)** The PPI network of yield-related genes in rice. **(B)** The PPI network of yield-related genes in maize.

Here, the rice yield-related genes belonging to MADS-box, ARF, and AP2 domain-containing gene families were used to construct the genetic network. The AP2 domain-containing protein gene family contains nine proteins: OsEATB, OsRSR1, OsBBM1, OsBBM2, OsBBM3, OsSNB, OsIDS1, OsAP2-39, and OsERF078/FZP. There are three proteins, OsARF1, OsARF19/7a, and OsARF4/OsARF2, belonging to the ARF family. OsMADS1, OsMADS3, OsMADS7, OsMADS8, OsMADS13, and OsMADS17 belong to the MADS-box gene family. The protein sequences are provided in Supplementary File 5. The STRING database and Cytoscape_v3.7.2 were used to construct the protein-protein interaction network (PPI) (Szklarczyk et al., 2019) and the PPI network detail information is in Supplementary Table 3. From Figure 3A, there are 14 proteins from the three gene families, as well as seven proteins which have not been cloned. Among the seven proteins, OsqHd1, similar to SBP-domain protein 4, is a minor QTL with the functions of delaying heading and increasing the numbers of spikelets per panicle, grains per panicle and the grain yield per plant (Chen et al., 2014). *OsEBP-89*, *OsERF62*, *OsERF71*, *OsAP2-20*, and *OsAP2-37* indicated that the AP2 domain-containing gene family plays an important role in rice yield. NRPB3-like (*Os09g0110400*) and *OMTN4* (*Os06g0675600*) belong to the NAC gene family, and overexpression of *OMTN4* negatively affected drought tolerance during the rice reproductive stage (Fang et al., 2014). The homologous gene for ABC1-like (Os01g0318700) protein is *AtOSA1* (AT4G01660) in Arabidopsis, a member of the ATH subfamily which encodes an ABC transporter (Jasinski et al., 2008).

### Prediction and Analysis of Genetic Network for Maize Yield-Related Genes

Some yield-related genes were randomly selected from the MADS-box, ARF and AP2 domain-containing gene families in maize. The AP2 domain-containing protein gene family contains three proteins, ZmEREB94, ZmEREB156 and ZmRap2.7, as well as ZmMADS1, ZmMADS3, ZmMADS31, Zmm4, ZmMADS1a, and ZmMADS2, which belong to the MADS-box gene family. The protein sequences are provided in Supplementary File 5. The STRING database and Cytoscape_v3.7.2 were used to construct the protein-protein interaction network (PPI) (Szklarczyk et al., 2019) and the PPI network detail information is in Supplementary Table 3. From Figure 3B, there are seven cloned proteins from the three gene families and 21 unknown proteins. Among the 21 proteins, ZmMYBR66 and ZmMYBR106 belong to the MYB gene family, Zm00001d002684 has the function of flower locus D, and Zmhda108 and ZmHDA19 belong to the histone deacetylase family. A large number of uncloned genes were uncharacterized proteins.

### Analysis of the Similar Genetic Network for Maize and Rice Yield-Related Genes

**FIGURE 4:**
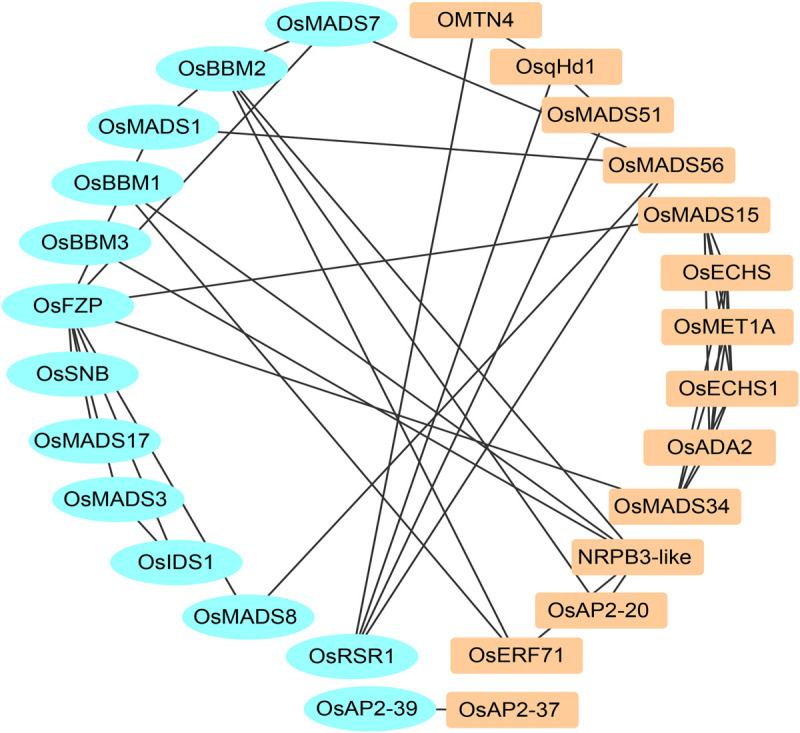
The PPI network of rice and maize yield-related genes.

In this research, the yield-related genes were selected from total rice and maize in Supplementary Table 4 and Supplementary File 5. Twenty-eight protein sequences containing 18 rice protein sequences and 10 maize protein sequences were used to construct the co-network using the STRING database. Based on the previous research, there are 13 cloned proteins that interact with the other 18 new proteins (Figure 4).

*OsERF71*, *OsAP2-20*, *OsAP2-37*, *NRPB3-like* (Os09g0110400), and *OMTN4* (Os06g0675600) suggested that the AP2 domain-containing and NAC gene family serve important roles in rice yield. *OsqHd1* also takes part in increasing the number of grains per panicle and grain yield (Chen et al., 2014). Overexpression of *OsMADS56* also resulted in delayed flowering in the situation of long days (Ryu et al., 2009). There is an aberrant phenotype of the mutant o*smads34-t* compared with the wild plants, such as more primary branch numbers, abnormal panicles, and the length of sterile lemmas: therefore, *OsMAD*S34 is involved in rice yield and grain size (Kobayashi et al., 2009, 2012; Gao et al., 2010; Yu et al., 2016). Loss of *OsMADS51* exerted little effect on flowering in long days, and *OsMADS51* can transmit an SD promotion signal from *OsGI* to *Ehd1* as a novel flowering promoter (Kim et al., 2008). The axillary buds exhibited accelerated development and frequently grew into effective tillers upon overexpression of *OsMADS15*. The panicles were large in the *OsMADS15* transgenic rice (Lu et al., 2012). OsMET1A is a DNA methyltransferase which is primarily responsible for maintaining CpG methylation (Yamauchi et al., 2008). OsECHS, OsECHS1 and OsADA2 are three uncharacterized proteins. These results suggested that most proteins in the predicted network are yield-related genes.

## Discussion

A large diversity of agronomic traits are important determinants of yield in rice and maize, such as grain size, shape weight, spikelets and tillers per plant, among others. Brassinolide (BR) and auxin, as the most important plant hormones, serve important roles in grain development and regulate factors such as grain size, shape, and weight.

Maize and rice share a common ancestor. Similar traits were usually controlled by QTLs in syntenic regions among species. Many genes that may affect seed shape and weight have been mapped and cloned in rice, such as *GS3*, *GW2*, and *GS5*. *GS3* and *GW2* were isolated for maize orthologs genes of rice, and found that maize genes also controlled similar traits. AP2 domain, ARF domain, and MADS genes families are involved in the determinants of yield. Previous studies have suggested that AP2 functions as a nuclear transcription factor in plant cells. Auxin and BR serve important roles in the development of seeds and genes (Zuo and Li, 2014). The ARF family members function as transcriptional activators and repressors in plants (Guilfoyle, 2015). In addition, *ARF18* impacts the expression of the downstream auxin-responsive genes and affects silique length and seed weight (Liu et al., 2015b). However, previous research indicates that rice grain weight, grain size, grain hull, endosperm development and activity in maternal tissues are negatively regulated by *OsARF4* (Hu et al., 2018). In addition, WRINKLED1 (WRI1), as the AP2/EREBP transcription factor in Arabidopsis, also serves the function of seed storage metabolism (Maeo et al., 2009). In rice, OsERF2 mediated gene expression in the metabolism of sucrose and plant hormone signaling pathways affecting the accumulation of sucrose and UDPG (Maeo et al., 2009). The MADS family is a group of crucial regulatory factors that control the development of floral organs: for example, the *OsMADS1* gene can induce flowering (Chung et al., 1994).

In our study, there were several genes predicted to participate in the yield-related network, and these genes were uncharacterized genes belonging to diverse gene families. Some genes were known as yield-related genes, OsqHd1, an SBP-domain protein, serves the function of delaying heading and increasing grain yield. NRPB3-like was a predicted gene of the NAC gene family, and ABC1-like was an aarF domain-containing protein kinase. These genes are novel, so further studies on the functions of these unknown genes are necessary.

## Data Availability Statement

The original contributions presented in the study are included in the article/Supplementary Material, further inquiries can be directed to the corresponding authors.

## Author Contributions

QZ designed the research. ZC and ZS performed the research and wrote the manuscript. ZC, ZS, and DZ analyzed the data. All authors read and approved the manuscript.

## Conflict of Interest

The authors declare that the research was conducted in the absence of any commercial or financial relationships that could be construed as a potential conflict of interest.

## Footnotes

1 http://www.ricedata.cn/gene/

2 https://string-db.org/

3 https://www.ncbi.nlm.nih.gov/
