# Supplementary material for: Regulator Network Analysis of Rice and Maize Yield-Related Genes
Source: Front Cell Dev Biol. 2020 Dec 3;8:621464. doi: 10.3389/fcell.2020.621464 (PMC7793993; doi:10.3389/fcell.2020.621464)
Supplement: Supplementary file 1 [file Data_Sheet_1.docx]

**Supplementary material 5 Sequences information for PPI network construction**

**Rice**

| **>OSMADS3** |
| --- |
| **LAYPSMMNMMTDLSCGPSSMTELTAAAAPAGSGSSAAVAAGSSEKMGRGKIEIKRIENTTNRQVTFCKRRNGLLKKAYELSVLCDAEVALIVFSSRGRLYEYANNSVKSTVERYKKANSDTSNSGTVAEVNAQHYQQESSKLRQQISSLQNANSLTRLA** |
| **>OsMADS13** |
| **MGRGRIEIKRIENTTSRQVTFCKRRNGLLKKAYELSVLCDAEVALIVFSSRGRLYEYSNNNNVKATIDRYKKAHACGSTSGAPLIEVNAQQYYQQESAKLRHQIQMLQNTNKHLVGDNVSNLSLKELKQLESRLEKGISKIRARKNELLASEINYMAKREIELQNDNMDLRTKIAEEEQQLQQVTVARSAAMELQAAAAAQQQQQNPFAVAAAQLDMKCFFPLNLFEAAAQVQAVAAQRQQIIPTELNLGYHHHLAIPGAAAADAPPPHF** |
| **>OsMADS7** |
| **MGRGRVELKRIENKINRQVTFAKRRNGLLKKAYELSVLCDAEVALIIFSNRGKLYEFCSTQSMTKTLEKYQKCSYAGPETAVQNRESEQLKASRNEYLKLKARVENLQRTQRNLLGEDLDSLGIKELESLEKQLDSSLKHVRTTRTKHLVDQLTELQRKEQMVSEANRCLRRKLEESNHVRGQQVWEQGCNLIGYERQPEVQQPLHGGNGFFHPLDAAGEPTLQIGYPAEHHEAMNSACMNTYMPPWLP** |
| **>OsMADS8** |
| **MGRGRVELKRIENKINRQVTFAKRRNGLLKKAYELSVLCDAEVALIIFSNRGKLYEFCSGQSMTRTLERYQKFSYGGPDTAIQNKENELVQSSRNEYLKLKARVENLQRTQRNLLGEDLGTLGIKELEQLEKQLDSSLRHIRSTRTQHMLDQLTDLQRREQMLCEANKCLRRKLEESNQLHGQVWEHGATLLGYERQSPHAVQQVPPHGGNGFFHSLEAAAEPTLQIGFTPEQMNNSCVTAFMPTWLP** |
| **>OsMADS1** |
| **MGRGKVELKRIENKISRQVTFAKRRNGLLKKAYELSLLCDAEVALIIFSGRGRLFEFSSSSCMYKTLERYRSCNYNSQDAAAPENEINYQEYLKLKTRVEFLQTTQRNILGEDLGPLSMKELEQLENQIEVSLKQIRSRKNQALLDQLFDLKSKEQQLQDLNKDLRKKLQETSAENVLHMSWQDGGGHSGSSTVLADQPHHHQGLLHPHPDQGDHSLQIGYHHPHAHHHQAYMDHLSNEAADMVAHHPNEHIPSGWI** |
| **>OsMADS17** |
| **MDRSEMGRGRVELKRIENKINRQVTFSKRRNGLLKKAYELSVLCDAEVALIIFSSRGKLYEFGSAGINKTLEKYNSCCYNAQGSNSALAGGEHQSWYQEMSRLKTKLECLQRSQRHMLGEDLGPLSIKELQQLEKQLEYSLSQARQRKTQIMMEQVDDLRRKERQLGELNKQLKNKLEAEADSSNCRSAIQDSWVHGTVVSGGRVLNAQPPPDIDCEPTLQIGYYQFVRPEAANPRSNGGGGDQNNNFVMGWPL** |
| **>OsARF1** |
| **MSSQGAGGGVGDPELFAELWRACAGPLVEVPQRDERVFYFLQGHLEQLQEPTDPALLAEQIKMFQVPYKILCKVVNVELKAETETDEVFAQITLQPDPDQENLPTLPDPPLPEQPRPVVHSFCKILTPSDTSTHGGFSVLRRHANECLPPLDMSMATPTQELITKDLHGSEWRFKHIYRGQPRRHLLTTGWSTFVTSKKLISGDAFVYLRSETGEQRVGVRRLVQKQSTMPASVISSQSMHLGVLASASHAIKTNSIFLVYYRPRLSQSQYIVSVNKYLAASKVGFNVGMRFKMSFEGEDVPVKKFSGTIVGEGDLSLQWSGSEWKSLKVQWDEVTNVNGPERVSPWEIETCDGTAPAINVPLQSATKNKRPREPSETIDLQSLEPAQEFWLSGMPQQHEKTGIGSSEPNCISGHQVVWPGEHPGYGAVSSSVCQNPLVLESWLKDFNSSNKGVSPTLSEISQKIFQVTSNEARIATWPARSAYQAEEPTSKLSSNTAACGYRTEEVAPNASKVVEGKKEPAMFRLFGVDLMKCTSISTTTDDKSSVGAGEASAKGTGSHEDSGQLSAFSKVTKEHIAADESPQEIQSHQNYTARTRIKVQMHGNAVGRAVDLANLDGYEQLMNELEEMFNIKDLKQKWKVAFTDDEGDTMEVGDDPWLEFCQMVRKIVLYPIEDEKKIEPHPKLLSSANPEQDQKTGF** |
| **>OsARF19/OsARF7a** |
| **MMKQAQQQPPPPPASSAATTTTAMAAAAAAAVVGSGCEGEKTKAPAINSELWHACAGPLVSLPPAGSLVVYFPQGHSEQVAASMQKDVDAHVPSYPNLPSKLICLLHNVTLHADPETDEVYAQMTLQPVTSYGKEALQLSELALKQARPQTEFFCKTLTASDTSTHGGFSVPRRAAEKIFPPLDFSMQPPAQELQARDLHDNVWTFRHIYRGQPKRHLLTTGWSLFVSGKRLFAGDSVIFVRDEKQQLLLGIRRANRQPTNISSSVLSSDSMHIGILAAAAHAAANNSPFTIFYNPRASPTEFVIPFAKYQKAVYGNQISLGMRFRMMFETEELGTRRYMGTITGISDLDPVRWKNSQWRNLQVGWDESAAGERRNRVSIWEIEPVAAPFFICPPPFFGAKRPRQLDDESSEMENLLKRAMPWLGEEICIKDPQTQNTIMPGLSLVQWMNMNMQQSSSFANTAMQSEYLRSLSNPNMQNLGAADLSRQLCLQNQLLQQNNIQFNTPKLSQQMQPVNELAKAGIPLNQLGVSTKPQEQIHDASNLQRQQPSMNHMLPLSQAQTNLGQAQVLVQNQMQQQHASSTQGQQPATSQPLLLPQQQQQQQQQQQQQQQQQQQQKLLQQQQQQLLLQQQQQLSKMPAQLSSLANQQFQLTDQQLQLQLLQKLQQQQQSLLSQPAVTLAQLPLIQEQQKLLLDMQQQLSNSQTLSQQQMMPQQSTKVPSQNDGLLHQNFPMSNFNQPQMFKDAPPDAEIHAANTSNNALFGINGDGPLGFPIGLGTDDFLSNGIDAAKYENHISTEIDNSYRIPKDAQQEISSSMVSQSFGASDMAFNSIDSTINDGGFLNRSSWPPAAPLKRMRTFTKVYKRGAVGRSIDMSQFSGYDELKHALARMFSIEGQLEERQRIGWKLVYKDHEDDILLLGDDPWEEFVGCVKCIRILSPQEVQQMSLEGCDLGNNIPPNQACSSSDGGNAWRARCDQNSGNPSNGSYEQFE** |
| **>OsARF4/OsARF2** |
| **MPPAAMAPPPPPQGSSTGDPLYDELWHACAGPLVTVPRVGDLVFYFPQGHIEQVEASMNQVADSQMRLYDLPSKLLCRVLNVELKAEQDTDEVYAQVMLMPEPEQNEMAVEKTTPTSGPVQARPPVRSFCKTLTASDTSTHGGFSVLRRHADECLPPLDMTQSPPTQELVAKDLHSMDWRFRHIFRGQPRRHLLQSGWSVFVSSKRLVAGDAFIFLRGENGELRVGVRRAMRQLSNVPSSVISSQSMHLGVLATAWHAINTKSMFTVYYKPRTSPSEFIIPYDQYMESVKNNYSVGMRFRMRFEGEEAPEQRFTGTIIGSENLDPVWPESSWRSLKVRWDEPSTIPRPDRVSPWKIEPASSPPVNPLPLSRVKRPRPNAPPASPESPILTKEAATKVDTDPAQAQRSQNSTVLQGQEQMTLRSNLTESNDSDVTAHKPMMWSPSPNAAKAHPLTFQQRPPMDNWMQLGRRETDFKDVRSGSQSFGDSPGFFMQNFDEAPNRLTSFKNQFQDQGSARHFSDPYYYVSPQPSLTVESSTQMHTDSKELHFWNGQSTVYGNSRDRPQNFRFEQNSSSWLNQSFARPEQPRVIRPHASIAPVELEKTEGSGFKIFGFKVDTTNAPNNHLSSPMAATHEPMLQTPSSLNQLQPVQTDCIPEVSVSTAGTATENEKSGQQAQQSSKDVQSKTQVASTRSCTKVHKQGVALGRSVDLSKFSNYDELKAELDKMFEFDGELVSSNKNWQIVYTDNEGDMMLVGDDPWEEFCSIVRKIYIYTKEEVQKMNSKSNAPRKDDSSENEKGHLPMPNKSDN** |
| **>OsEATB** |
| **MTKKVIPAMAAARQDSCKTKLDERGGSHQAPSSARWISSEQEHSIIVAALRYVVSGCTTPPPEIVTVACGEACALCGIDGCLGCDFFGAEAAGNEEAVMATDYAAAAAAAAVAGGSGGKRVRRRRKKNVYRGVRHRPWGKWAAEIRDPRRAVRKWLGTFDTAEEAARAYDRAALEFRGARAKLNFPCSEPLPMPSQRNGNGGDAVTAATTTAEQMTPTLSPCSADAEETTTPVDWQMGADEAGSNQLWDGLQDLMKLDEADTWFPPFSGAASSF** |
| **>OsRSR1** |
| **MELDLNNVAEGVVEKHETAARSDSGTSESSVLNGEASGAAIAPAEEGSSSTPPSPPPPPAAVLEFSILRSSASASGENDADDDEEEEATPSPPPHHQHQQLLVTRELFPSAAPSPQHWAELGFLRPDPPRPHPDIRILAHAPPPAPPPPPPQPQPQAAKKSRRGPRSRSSQYRGVTFYRRTGRWESHIWDCGKQVYLGGFDTAHAAARAYDRAAIKFRGVEADINFNLSDYEEDMRQMKSLSKEEFVHVLRRQSTGFSRGSSKYRGVTLHKCGRWEARMGQFLGKKYIYLGLFDSEVEAARAYDKAAIKCNGREAVTNFEPSTYDGELPTDAAAQGADVDLNLRISQPAASQQSPKRDSGSLGLQIHHGSFEGSEFKRAKNDAAPSELASRPHRFPLLTEHPPIWTAQPHPLFPNNEDASRSSDQKRKPSEGVAVPSWAWKQVSHHHPAPPHTLPLPFFSSSSSSPSSSSAAASSGFSKAATTAAAAQHTATLRFDPTAPSSSSSSRHHHHH** |
| **>OsBBM3** |
| **MATMNNWLAFSLSPQDQLPPSQTNSTLISAAATTTTAGDSSTGDVCFNIPQDWSMRGSELSALVAEPKLEDFLGGISFSEQQHHHGGKGGVIPSSAAACYASSGSSVGYLYPPPSSSSLQFADSVMVATSSPVVAHDGVSGGGMVSAAAAAAASGNGGIGLSMIKNWLRSQPAPQPAQALSLSMNMAGTTTAQGGGAMALLAGAGERGRTTPASESLSTSAHGATTATMAGGRKEINEEGSGSAGAVVAVGSESGGSGAVVEAGAAAAAARKSVDTFGQRTSIYRGVTRHRWTGRYEAHLWDNSCRREGQTRKGRQVYLGGYDKEEKAARAYDLAALKYWGPTTTTNFPVNNYEKELEEMKHMTRQEFVASLRRKSSGFSRGASIYRGVTRHHQHGRWQARIGRVAGNKDLYLGTFSTQEEAAEAYDIAAIKFRGLNAVTNFDMSRYDVKSILDSAALPVGTAAKRLKDAEAAAAYDVGRIASHLGGDGAYAAHYGHHHHSAAAAWPTIAFQAAAAPPPHAAGLYHPYAQPLRGWCKQEQDHAVIAAAHSLQDLHHLNLGAAAAAHDFFSQAMQQQHGLGSIDNASLEHSTGSNSVVYNGDNGGGGGGYIMAPMSAVSATATAVASSHDHGGDGGKQVQMGYDSYLVGADAYGGGGAGRMPSWAMTPASAPAATSSSDMTGVCHGAQLFSVWNDT** |
| **>OsBBM2** |
| **MTRQEYIAYLRRNSSGFSRGASKYRGVTRHHQHGRWQARIGRVAGNKDLYLGTFSTEEEAAEAYDIAAIKFRGLNAVTNFDMSRYDVKSILESSTLPVGGAARRLKEAADHAEAAGATIWRAADMDGAGVISGLADVGMGAYAASYHHHHHHGWPTIAFQQPPPLAVHYPYGQAPAAPSRGWCKPEQDAAVAAAAHSLQDLQQLHLGSAAAHNFFQASSSSTVYNGGGGGYQGLGGNAFLMPASTVVADQGHSSTATNHGNTCSYGNEEQGKLIGYDAMAMASGAAGGGYQLSQGSASTVSIARANGYSANWSSPFNGAMG** |
| **>OsBBM1** |
| **MASITNWLGFSSSSFSGAGADPVLPHPPLQEWGSAYEGGGTVAAAGGEETAAPKLEDFLGMQVQQETAAAAAGHGRGGSSSVVGLSMIKNWLRSQPPPAVVGGEDAMMALAVSTSASPPVDATVPACISPDGMGSKAADGGGAAEAAAAAAAQRMKAAMDTFGQRTSIYRGVTKHRWTGRYEAHLWDNSCRREGQTRKGRQVYLGGYDKEEKAARAYDLAALKYWGTTTTTNFPVSNYEKELDEMKHMNRQEFVASLRRKSSGFSRGASIYRGVTRHHQHGRWQARIGRVAGNKDLYLGTFGTQEEAAEAYDIAAIKFRGLNAVTNFDMSRYDVKSIIESSNLPIGTGTTRRLKDSSDHTDNVMDINVNTEPNNVVSSHFTNGVGNYGSQHYGYNGWSPISMQPIPSQYANGQPRAWLKQEQDSSVVTAAQNLHNLHHFSSLGYTHNFFQQSDVPDVTGFVDAPSRSSDSYSFRYNGTNGFHGLPGGISYAMPVATAVDQGQGIHGYGEDGVAGIDTTHDLYGSRNVYYLSEGSLLADVEKEGDYGQSVGGNSWVLPTP** |
| **>OsSNB** |
| **MVLDLNVESPGGSAATSSSSTPPPPPDGGGGGYFRFDLLGGSPDEDGCSSPVMTRQLFPSPSAVVALAGDGSSTPPLTMPMPAAAGEGPWPRRAADLGVAQSQRSPAGGKKSRRGPRSRSSQYRGVTFYRRTGRWESHIWDCGKQVYLGGFDTAHAAARAYDRAAIKFRGLDADINFNLNDYEDDLKQMRNWTKEEFVHILRRQSTGFARGSSKYRGVTLHKCGRWEARMGQLLGKKYIYLGLFDSEIEAARAYDRAAIRFNGREAVTNFDPSSYDGDVLPETDNEVVDGDIIDLNLRISQPNVHELKSDGTLTGFQLNCDSPEASSSVVTQPISPQWPVLPQGTSMSQHPHLYASPCPGFFVNLREVPMEKRPELGPQSFPTSWSWQMQGSPLPLLPTAASSGFSTGTVADAARSPSSRPHPFPGHHQFYFPPTA** |
| **>OsIDS1** |
| **MLLDLNVESPERSGTSSSSVLNSGDAGGGGGGGGGGGLFRFDLLASSPDDDECSGEQHQLPAASGIVTRQLLPPPPPAAPSPAPAWQPPRRAAEDAALAQRPVVAKKTRRGPRSRSSQYRGVTFYRRTGRWESHIWDCGKQVYLGGFDTAHAAARAYDRAAIKFRGLEADINFNLSDYEDDLKQMRNWTKEEFVHILRRQSTGFARGSSKFRGVTLHKCGRWEARMGQLLGKKYIYLGLFDTEVEAARAYDRAAIRFNGREAVTNFEPASYNVDALPDAGNEAIVDGDLDLDLRISQPNARDSKSDVATTGLQLTCDSPESSNITVHQPMGSSPQWTVHHQSTPLPPQHQRLYPSHCLGFLPNLQERPMDRRPELGPMPFPTQAWQMQAPSHLPLLHAAASSGFSAGAGAGVAAATRRQPPFPADHPFYFPPTA** |
| **>OsAP2-39** |
| **MAPRNAAEAVAVAVAEGGGAGMEPRFRGVRKRPWGRYAAEIRDPARKARVWLGTFDTAEAAARAYDSAALHFRGPKAKTNFPVAFAHAHHHAPPPPLPKAAALAVVSPTSSTVESSSRDTPAAAPVAAAAKAQVPASPSLDLSLGMSAMVAAQPFLFLDPRVAVTVAVAAPVPRRPAVVSVKKEVARLDEQSDTGSSSSVVDASPAVGVGLDLNLPPPIEEA** |
| **>OsERF078/FZP** |
| **MNTRGSGSSSSSSSSQASLMAFSEPPKPASQPSPPSSPMSERPPSGRSRRRAQEPGRFLGVRRRPWGRYAAEIRDPTTKERHWLGTFDTAQEAALAYDRAALSMKGAQARTNFVYTHAAYNYPPFLAPFHAPQYAAAAAAPSSVQYGGGVGAAPHIGSYGHHHHHHHHHGHGAASGASSVGECSTMPVMVPVDPHRSSMSSSLLDMDRNGHDFLFSGADDNSGYLSSVVPESCLRPRGGGAAADHQDMRRYSDADAYGMMGLREDVDDLAQMVAGFWGGGDAADQLGACGFPASGGAADMVASSQGSDSYSPFSFLSH** |

**Maize**

| **>ZmEREB156** |
| --- |
| **MAPLAAAAVKMEAEQAAMAAPQLGAAHQQTQPRRQYRGVRMRKWGKWVAEIREPHKRTRIWLGSYATAVAAARAYDTAVFYLRGRSARLNFPEEIPSFGLADGVDVGEHARDPAAAAAGGGGGCTLSAASIRKKAIEVGSRVDALQTGMVVPPPHHRERHRHHNHLPQLRVHAEEQQEEEEQKPQRPAWSGRVKNPDLNRAPSPESSDAE** |
| **>ZmEREB94** |
| **MSPALLPLHGSELTSSASSSCSFLQPGQGGARDCGFRFRISGCKYNTNMEVAMTQLINFLPCTALASYFAAPEIVKEFKNPPQITVSLGSHLWSIWKRCVKMRPSVQKVRIFCSDPDATDSSDDEDGQIIAVKKMVMEILVPVTNSKTSNSLKTLVPCGAKDLEVSEKKGKSSRFRGVRRRRWGRWAAEIRDPVRKTRKWIGSYDSEEAAAAAYQAYAKQIREELLAIKNQRSVSERAALSSSSSVSCVSSSPPCEQTGHEPQTRVLVEKDPEPVDEVFLNFSLTPKGISMDDLLGRIDEIPVCDDPVSPTDELPLDNFTSLEDAFPISDFIGSRDEHLYEHYIGLADISHLPLPMDDPAFNLDAELDWSGFDFAAIEGELDVL** |
| **>ZmMADS1** |
| **MGRGRIEIKRIENNTSRQVTFCKRRNGLLKKAYELSVLCDAEVALVVFSSRGRLYEYANNSVKATIERYKKAHAVGSSSGPPLLEHNAQQFYQQESAKLRNQIQMLQNTNRLFICQTLIAEFLFQSELLAAEINYMAKRETELQNDHMNLRTKIEEGEQQLQQVTVAQSVAAAAATDVELNPFLEMDTKCFFPGGPFATLDMKCFFPGSLQMLEAQQRQMLATELNLGYQLAPPDTDVANNNPQQF** |
| **>ZmMADS69** |
| **MAPRGRVELRRIEDKASRQVRFSKRRAGLFKKAFELALLCDAEVALLVFSPGGKLYEYSSSRFRTPTPSS** |
| **>ZmRap2.7** |
| **MQLDLNVAEAPPPVEMEASDSGSSVLNASEAASAGGAPAPAEEGSSSTPAVLEFSILIRSDSDAAGADEDEDATPSPPPRHRHQHQQQLVTRELFPAGAGPPAPTPRHWAELGFFRADLQQQQAPGPRIVPHPHAAPPPAKKSRRGPRSRSSQYRGVTFYRRTGRWESHIWDCGKQVYLVVVRLLADIRRWIRHRSRRCKARTGLDAQNSSAYDRAAIKFRGVDADINFNLSDYEDDMKQMGSLSKEEFVHVLRRQSTGFSRGSSRYRGVTLHKCGRWEARMGQFLGKKYIYLGLFDSEVEAARAYDKAAIKCNGREAVTNFEPSTYHGELPTEVADVDLNLSISQPSPQRDKNSCLGLQLHHGPFEGSELKKTKASANDRYTLTS** |
| **>ZmMADS3** |
| **MGRGKVQLKRIENKINRQVTFSKRRNGLLKKAHEISVLCDAEVAVIVFSPKGKLYEYASDSRGRWLSPDY** |
| **>ZmMADS31** |
| **MQNNYQEYVKLKARVEVLQHSQRNLLGEELAPLSPSELDQLESQVDKTLKQIRSRKTQVLLDELCDLKRKEQMLQDANRVLKRKLHEFEAEAASPPQLAWQGGGGMLSHDPPQPEHFFVALESNAPLQPT** |
| **>Zmm4** |
| **MSGSNWLKNDRLCPVFAASSLWQQAGGWWKGQQRSRRLALDCHHGCLAI** |
| **>ZmMADS1a** |
| **MVRGKTQMKRIENPTSRQVTFSKRRNGLLKKAFELSVLCDAEVALVVFSPRGKLYEFASGSAQKTIERYRTYTKDNVSNKTVQQDIERVKADADGLSKRLEALEAYKRKLLGERLEDCSIEELHSLEVKLEKSLHCIRGRKTELLEEQVRKLKQKEMSLRKSNEDLREKCKKQPPVPMASAPPRAPAVDNVEDGHREPKDDGMDVETELYIGLPGRDYRSSKDKAAVAVRSG** |
| **>ZmMADS2** |
| **MGRGKIVIRRIDNSTSRQVTFSKRRNGIFKKAKELAILCDAEVGLVIFSSTGRLYEYSSTSMKSVIDRYGKAKEEQQVVANPNSELKFWQREAASLRQQLHNLQENYRQLTGDDLSGLNVKELQSLENQLETSLRGVRAKKDHLLIDEIHDLNRKASLFHQENTDLYNKINLIRQENDELHKKIYETEGPSGVNRESPTPFNFAVVETRDVPVQLELSTLPQQNNIEPSTAPKLGLQLIP** |
| **>ZmFZP** |
| **MADQAVAATTAHDQHQQQQRRRRSRASSEYLGVRRRPWGRYAAEIRNPVTKERHWLGTFDTAEDAAVAYDLSAISISGPAAARTNFCYPRGAARGLPSAHALPRQGLQLLPHSPAVPSPAAPPSALLPAGGGGGGGTVDGCDYDCEWRHLKAVEEADADDESMTIAAILQSFQHLNAPSAPPASLY** |
